# Molluscicidal Activity of Methomyl and Cardenolide Extracts from *Calotropis procera* and *Adenium arabicum* Against the Land Snail *Monacha cantiana*

**DOI:** 10.3390/molecules17055310

**Published:** 2012-05-07

**Authors:** Ali Al-Sarar, Hamdy Hussein, Yasser Abobakr, Alaa Bayoumi

**Affiliations:** Plant Protection Department, College of Food and Agriculture Sciences, King Saud University, P.O. Box 2460, Riyadh 11451, Saudi Arabia; Email: hhussein1@ksu.edu.sa (H.H.); ymohamed@ksu.edu.sa (Y.A.); amostafa1@ksu.edu.sa (A.B.)

**Keywords:** molluscicides, *Monacha cantiana*, *Adenium arabicum*, *Calotropis procera*, cardenolides, land snails

## Abstract

In this work, we have evaluated the molluscicidal activity of two cardenolide extracts from *Adenium arabicum* Balf f. [the benzene (B) and methanol (M) extracts], one cardenolide extract from *Calotropis procera* (Aiton) W.T. Aiton (extract C), and methomyl against the harmful land snail *Monacha cantiana* (Montagu). The contact LD_50_ values for the above mentioned plant extracts were 12.62, 34.63, and 34.35 mg·kg^−1^ of body weight, respectively, while the LD_50_ for methomyl was 116.62 mg·kg^−1^, that is, the plant extracts were 9.24, 3.37, and 3.4 times more toxic than methomyl. In addition, a simple colorimetric method, based on Kedde reagent, was modified to determine cardenolide concentrations in plant extracts. Thin layer chromatography analysis (TLC) showed several cardiac glycosidal compounds in each plant extract. The results proved that cardiac glycosides are promising candidate compounds that could be used to control land snails, or exploited to develop new, effective, and environmentally friendly molluscicides.

## 1. Introduction

Land mollusks (snails and slugs), including *Monacha cantiana*, are very harmful pests to fruit trees, vegetables, field crops, ornamentals and ecosystem [1,2]. Metaldehyde and few carbamates are the most used pesticides to control these pests in the form of toxic baits. These pesticides are hazardous to the environment and non-target organisms that come in contact with/ingest these toxic baits [3,4,5,6,7]. Moreover, the efficacy of these molluscicides declines rapidly in moist conditions [8]. In our opinion, the worst disadvantage of these molluscicides is the high concentration of active ingredients (0.5–5%) required to render their baits effective against target pests [9,10]. Methomyl and methiocarb, although highly toxic to mammals and insects, are moderately toxic to land snails [11,12,13]. To overcome these problems, there is a need for new, more effective and less hazardous molluscicides. In previous studies, we have discovered the promising molluscicidal properties of uscharin from *Calotropis procera*, ouabin from *Acokanthera ouabaio *Lewin, cardenolide extracts from *Pergularia tomentosa* Linn. and *Nerium oleander* L. [11,14,15,16]. Also, *Thevetia peruviana* (Pers.) K. Schum. (contains cardenolides) was found to be toxic to slug and snail pests [17]. Previous studies have shown that *Adenium arabicum* (previously *A. obesum*) contains cardenolides [18], so this work was carried out to see if *A. arabicum*, grown in Saudi Arabia, displayed molluscicidal properties. In addition, based on successful preliminary trials, a simple colorimetric method, based on the Kedde reagent, was modified to determine the concentration of cardenolides in the plant extracts to follow the progress and successful isolation of the active compounds.

## 2. Results

### 2.1. Contact Toxicity

The results of contact toxicity of the benzene (B) and methanol (M) extracts from *A. arabicum*, as well as *C. procera* (C) extract against adult *M. cantiana* are presented in Table 1 and Table 2. In the case of extract B, the range of doses required to achieve 13.3–96.7% mortality was narrow (10–20 mg·kg^−1^), the LD_50_ value for this extract was 12.62 mg·kg^−1^. Significant differences (*p* < 0.05) were found between all test doses and the control group, except at the lowest dose. The results showed a significant increase in mortality percent (*p* < 0.05) between the two doses, 12 and 16 mg·kg^−1^. In the case of extract M, higher doses were needed to get the Ldp line (32–48 mg·kg^−1^); the LD_50_ value for this extract was 34.63 mg·kg^−1^. A significant difference in mortality percent was found between all test doses and the control group; furthermore, the differences in mortality were significant among all test doses. Although extract C required a higher range of doses (30–80 mg·kg^−1^) to achieve the required mortality percentages (33.3–100%), it showed almost the same toxicity as extract M (the LD_50_ was 34.35 mg·kg^−1^), Table 2.

Significant differences (*p* < 0.05) were found between all test doses and the control group. The 60 mg·kg^−1^ treatment showed a significant difference in percent mortality compared with the 40 mg·kg^−1^ treatment, but the difference in mortality between the 60 and 80 mg·kg^−1^ treatments was not significant. Methomyl was the least effective material tested against *M cantiana*, it showed LD_50_ value of 116.62 mg·kg^−1^.

**Table 1:** Average mortality values (% M) for extracts B, M, and C and methomyl after topical application to *Monacha cantiana*.

Extract B		Extract M		Extract C		Methomyl	
Dose *	% M	Dose *	% M	Dose *	% M	Dose *	% M
20	96.7 ^a^	48	93.3 ^a^	80	100.0 ^a^	200	93.3 ^a^
16	90.0 ^a^	42	70.0 ^b^	60	93.3 ^a^	160	60.0 ^b^
12	40.0 ^b^	36	56.7 ^c^	40	70.0 ^b^	120	46.7 ^b^^,c^
10	13.3 ^c^	32	40.0 ^d^	30	33.3 ^c^	80	40.0 ^c^
						60	3.3 ^d^
Control	0.0 ^c^	Control	0.0 ^e^	Control	0.0 ^d^	Control	0.0 ^d^
LSD	14.39	LSD	11.40	LSD	8.42	LSD	15.81

* Dose in mg·kg^−1^; Values followed by the same letter within a column are not significantly different at the 0.05 level. (LSD: Least Significant Difference).

**Table 2:** Probit analysis for contact toxicity of extracts B, M, and C and methomyl after topical application to *Monacha cantiana*.

Material	LD_50_ *	95% Fiducial limits	LD_ 95_	95% Fiducial limits	Slope ± SE
Extract B	12.62 ^a^	12.20–13.06	18.00	16.86–19.21	10.68 ± 0.78
Extract M	34.63 ^b^	33.22–36.10	53.28	48.70–58.30	8.79 ± 1.19
Extract C	34.35 ^b^	32.36–36.45	59.66	53.82–66.15	6.86 ± 0.49
Methomyl	116.62 ^c^	109.26–124.48	276.92	237.45–323.05	4.38 ± 0.13

* Values followed by the same letter within a column are not significantly different at the 0.05 level.

The 60 mg·kg^−1^ dose caused a negligible effect (only 3.3% mortality) and was not significantly different compared with the control treatment, while the 80 mg·kg^−1^ treatment resulted in 40% mortality, this treatment caused 100% mortality in the case of extract C. According to the LD_50_ values of tested materials, the extract B was the most effective material, followed by both the extract C and extract M, which were significantly less effective than the extract B. No significant difference was found between the effects of the C and M extracts. All three test extracts were significantly more effective than methomyl (Table 2).

### 2.2. Spectrophotometric Analysis

**Figure 1:**
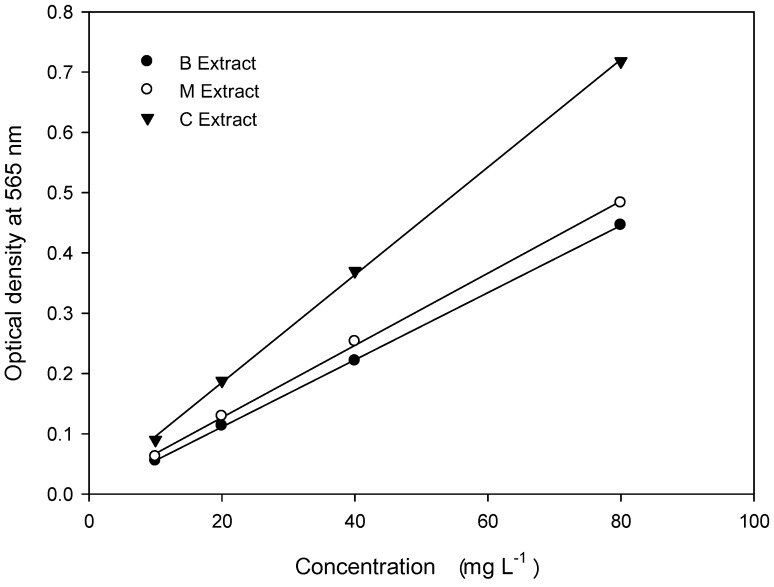
Relationship between the concentrations of B, M and C plant extracts and their corresponding optical densities at 565 nm.

The results of the spectrophotometric analysis of the cardenolide concentration in the B, M, and C plant extracts are shown in Table 3 and Figure 1.

**Table 3:** Optical densities* of extracts B, M, and C at 565 nm.

Conc. (mg·L^−1^)	Extract B *	Extract M	Extract C
10	0.055	0.062	0.09
20	0.113	0.129	0.188
40	0.221	0.253	0.37
80	0.446	0.483	0.718

* Every value is an average of two replicates.

All three extracts showed a good linear relationship between the concentration and optical density.

### 2.3. TLC Analysis

Analysis of the three plant extracts by TLC showed the presence of more than seven compounds in each extract, with some major, some medium, and some minor. The colors of the compounds were violet, red, or blue.

## 3. Discussion

Land snails cause great economic losses worldwide; however, there is no specific compound available to control these pests without being harmful to the non-target organisms. The highly toxic (to mammals and insects) organophosphorous and pyrethroid pesticides are not effective against land snails and are not used to combat them. Methomyl and methiocarb, carbamate pesticides, and metaldehyde are used to control land snails in the form of baits containing high concentrations of the active ingredients; however, these conventional pesticides have the previously mentioned disadvantages.

Cardenolide compounds or extracts are known to have strong molluscicidal activity against land snails [11,14,15,16]. The present study confirmed the above mentioned findings. All three cardenolide extracts were highly toxic to test snails, with extract B being the most effective one (LD_50_ = 12.62 mg·kg^−1^). It has been reported that the aqueous whole fruit extract of *Thevetia peruviana* is effective against the land snail *Achatina fulica* at ≥2% [17]; the lack of purification of the extract for the cardenolide reflects the poor molluscicidal activity. The authors sprayed the extract on to the target snails; they did not treat the animals individually at a certain mg·kg^−1^, as we did. Methomyl was moderately toxic (LD_50_ = 116.2 mg·kg^−1^) and was the least effective test material; many authors have shown that methomyl had moderate toxicity against land snails [11,13,19]. The highly toxic effect of methomyl to *Monacha obstructa* [14] could be attributed to the use of commercial methomyl (lannate 90%) with its additives and/or the higher sensitivity of the tested population. We have proposed that cardenolides may be leads for synthesis new, effective molluscicides and the mode of action of these compounds may be the base for biochemists to detect other simpler compounds with the same mode of action [14]. 

In addition to the possible uses of cardenolides [14], we think that seed dressings with cardenolide compounds (or extracts) could have the important economic and environmental advantages mentioned by Simms *et al.* [19]. In addition to their strong contact toxicity, cardenolides are strong antifeedants against land snails [20]; therefore, seed dressing with these materials could protect seeds and seedlings from snail attacks. Cardenolides are emetic to birds, and birds have learned to vomit and avoid prey that contain these compounds [21,22]; hence, seeds coated with cardenolides will be emetic and safe to birds. Furthermore, cardenolides are not toxic to insects that sequester these compounds from plants, and insects use these sequestered compounds as defensive tools against their predators [23,24]; therefore, seed dressing with these compounds is expected to be safer to beneficial carabid beetles (Carabidae) than seed dressing with conventional pesticides. The most interesting advantage of these compounds is their promising effect against cancer [24,25]. The mode of action of cardenolides in mammals is believed to be the inhibition of Na^+^-K^+^-ATPase; however, the Na^+^-K^+^-ATPase, isolated from *Theba pisana* snails, was insensitive to these compounds although the snails were very sensitive and killed by the treatment (Hussein HI, unpublished data); the target site of these compounds in land snails is still obscure. Methomyl, a carbamate pesticide, is used to control land snails and is known as a potent acetylcholinestersae inhibitor in insects and mammals, but we don’t think this enzyme is the main target for methomyl in land snails because organophosphorus pesticides, which are more potent inhibitors to acetylcholinestersae than carbamates, are not effective against land snails. Therefore, it is clear that the biochemistry and physiology of land snails differ from those of insects and mammals. 

Many reagents have been utilized for the quantitative determination of cardenolides [26,27], but these methods are not rapid and consume long time to obtain the maximum intensity of the developed color. In this respect, we think our present method is simple, precise, and saves time since it takes only about 3 min for the maximum intensity of color to develop and measured.

The results of TLC analysis of the three plant extracts (several compounds in each extract) agree well with previous studies. Thirty cardiac glycosides were isolated from *Adenium obesum* (recently *A. arabicum*) [18]; it is clear that we have separated these compounds into two groups, the less polar compounds (in the extract B) and the more polar compounds (in the extract M). Also, *C. procera* was shown to contain several cardiac glycosides [28,29].

## 4. Experimental

### 4.1. Test Snails

Test snails (*Monacha cantiana*) were collected from the soil and clover plants grown around date palm trees in a date palm farm, located in Riyadh, Saudi Arabia, in March and April, 2011. Snails were kept in plastic boxes under laboratory conditions (25 ± 2 °C), and provided with lettuce leaves to feed on.

### 4.2. Plant Extracts

#### 4.2.1. Extracts of *Adenium arabicum*

Stems of small *Adenium arabicum* trees (up to 25 cm height) were cut into small pieces (0.5–1 cm thickness) with the aid of pruning scissors and percolated with ethanol for 5 days at room temperature. After filtration and concentration under vacuum at 40 °C, aqueous ammonium sulfate (25%) was added and stirred with the ethanol extract. Precipitate was filtered and filtrate was extracted with 2 × 200 mL chloroform; the chloroform extract was stirred with a mixture of anhydrous sodium sulfate and charcoal and filtered. The filtrate was evaporated to give a viscous residue; this residue was treated with petroleum ether (60–80). The resulting residue was treated with benzene, and the remaining residue was dissolved in methanol. Evaporation of the benzene and methanol extracts to dryness gave crystallized B and M extracts, respectively. Both extracts B and M gave a strong positive reaction with Kedde reagent.

#### 4.2.2. *Calotropis procera* Extract

The *Calotropis procera* extract (extract C) used in this study was the same extract used in our previous work [30]. In brief, latex was mixed with ethanol; after filtration and concentration to a one third volume, the filtrate was treated with methanolic lead acetate (50%) to remove proteins, lipids, *etc.* in the usual way. The purified aqueous ethanol extract was shaken twice with chloroform; the chloroform extract was washed with sodium carbonate (5%) and distilled water and dried over anhydrous sodium sulfate. Solvent was evaporated to get the extract C. Identification of plants was carried out by the Botany Department, KSU, Saudi Arabia. 

### 4.3. Contact Toxicity

Contact toxicity tests were carried out according to our method [11]. All test materials were dissolved in the minimum volume of solvent (1% Tween 80 in ethanol) and diluted with water to get the required concentration; the highest concentration of ethanol was 10%. Preliminary trials were carried out to establish a suitable range of doses for each test material. Adult snails were used; every snail was weighed individually and received the volume that achieved the required dose according to its weight. For example, for a dose of 20 mg·kg^−1^, using a 0.2% solution of a test material, a 0.5 g snail received 5 µL of this solution, while a 0.35 g snail received 3.5 µL of the same solution. Doses were applied on the surface of the snail^'^s body inside the shell with the aid of an Eppendorf micropipette. Control treatments received solvent only. Three replicates were used for each dose, 10 animals each. After treatment, animals were transferred to 250 mL plastic cups, covered with cloth netting. After 24 h of treatment, dead animals were detected according to the method of WHO [31]; animals that did not respond when being touched with a pin inside the shell were considered dead. 

### 4.4. Spectrophotometric and TLC Analysis

#### 4.4.1. Spectrophotometric Analysis

A 5 N sodium hydroxide solution in distilled water, a 3,5-dinitrobenzoic acid solution in ethanol (2%) and a stock solution (1000 mg·L^−1^) from each extract in ethanol were prepared fresh. Volumes of 20, 40, 60, and 80 µL of the stock solution were mixed with 1.98, 1.96, 1.94, and 1.92 mL ethanol, respectively, in 15 mL glass test tubes; these solutions represented 10, 20, 40, and 80 mg·L^−1^ of the cardenolide extracts, respectively. One mL of the dinitrobenzoic acid solution was added to each tube, followed by 0.1 mL of the sodium hydroxide solution. After vortex, the maximum reading of the developed purple color was recorded at 565 nm, using ultraviolet-visible spectrophotometer (Shimadzu 1201, Shimadzu Corporation, Kyoto, Japan).

#### 4.4.2. TLC Analysis

Thin layer chromatography analysis was carried out using silica gel 60 precoated TLC plates (20 × 20 cm), from Merck. Mobile phase composed of ethylactate:methanol (97:3), and the detection was carried out by spraying with the chromogenic agent 3,5-dinitrobenzoic acid solution (2%), followed by a 20% sodium hydroxide solution.

### 4.5. Statistical Analysis

Analysis of results was conducted by using SAS software, all data were subjected to analysis of variance (ANOVA), and probit analysis was carried out according to Finney [32].

## 5. Conclusions

We can conclude that cardiac glycosides are very promising candidate compounds that could be used to control land snails, or exploited as a lead to develop new, effective, and environmentally friendly molluscicides. These compounds are effective against land snails and, in contrast to conventional pesticides, are less toxic to beneficial organisms. Land snails are affected through a very sensitive target that causes mortality within hours after exposure to these compounds; the mode of action of these compounds should be investigated. Individual compounds should be tested against many species of harmful land snails. The modified colorimetric method for determination of cardiac glycosides is simple and rapid and could help in many ways in the field of cardenolide research. 
